# Medical Programme

**Published:** 1883-01

**Authors:** 


### Medical Programme.

For January.

King's County Medical Society.—The annual meeting for the election of officers is to be held on the third Tuesday in January (the 16th), 1883.

New York County Medical Society.—Stated meeting, 22d January.

The Brooklyn Dental Society will hold iis next monthly meeting at the office of Dr. C. D. Cook, 133 Pacific avenue, on the 8th of January.

[Under this head we shall publish, each month, a programme of meetings of all the Medical and Dental Societies of this and neighboring cities. If the Secretaries of the Associations will send us one month in advance the date and place of meetings—regular, special and annual —we shall be happy to insert them here for the convenience of our readers. Hereafter the date of this programme will be made from the 15th of the month of issue to the 15th of the following month.]

